# Health Care Outcomes of Homelessness Prevention Programs in Veterans Experiencing Housing Instability

**DOI:** 10.1001/jamahealthforum.2025.6417

**Published:** 2026-01-23

**Authors:** Richard E. Nelson, Alec B. Chapman, Ann Elizabeth Montgomery, Ying Suo, Atim Effiong, Christa Shorter, Tom Greene, Jack Tsai, Lillian Gelberg, Stefan G. Kertesz, Thomas Byrne

**Affiliations:** 1 IDEAS Center, Veterans Affairs Salt Lake City Health Care System, Salt Lake City, Utah; 2 Department of Internal Medicine, University of Utah School of Medicine, Salt Lake City; 3 VA National Center on Homelessness Among Veterans; 4 Department of Population Health Sciences, University of Utah School of Medicine, Salt Lake City; 5 Birmingham, Alabama VA Health Care System, Birmingham; 6 School of Public Health, University of Alabama at Birmingham; 7 School of Public Health, University of Texas Health Sciences Center, San Antonio; 8 Department of Family Medicine, David Geffen School of Medicine at UCLA, Los Angeles, California; 9 Office of Healthcare Transformation and Innovation, VA Greater Los Angeles Healthcare System, Los Angeles, California; 10 Department of Health Policy & Management, Fielding School of Public Health, UCLA, Los Angeles, California; 11 UAB Heersink School of Medicine, Birmingham, Alabama; 12 School of Social Work, Boston University, Boston, Massachusetts; 13 Center for Healthcare Organization and Implementation Research, Bedford VA Medical Center, Bedford, Massachusetts

## Abstract

**Importance:**

Homelessness is associated with negative health outcomes and increased health care costs. The United States Department of Veterans Affairs (VA) Supportive Services for Veteran Families (SSVF) program provides housing-related financial assistance and other supports to veterans experiencing housing instability; however, little is known regarding short-term assistance interventions with a prevention focus.

**Objective:**

To estimate potential impacts of the SSVF program in mortality and health care cost outcomes over 3 years following program entry.

**Design, Setting, and Participants:**

Using observational data, outcomes were compared between veterans who enrolled in SSVF with those who did not for each month from October 2015 to December 2018. A propensity score for SSVF enrollment was calculated using observable characteristics including demographics, housing history, health care cost history, comorbidities, and geography. Using inverse probability of treatment weighting—a propensity score–based method that creates a pseudopopulation in which treatment groups are balanced on observed covariates—the potential impacts of SSVF enrollment in mortality were estimated using a Cox proportional hazards regression and health care costs with a generalized linear model over the 3 years following the trial index date. Data were from the VA electronic health record for a cohort of veterans receiving care in the VA system. Each trial drew on veterans with evidence of homelessness in structured and unstructured medical records during the previous month. Data were analyzed from November 1, 2023, to September 9, 2025.

**Exposure:**

The exposure was enrollment in the SSVF program, from the Homeless Management Information System data.

**Main outcome:**

The main outcomes were all-cause mortality and VA health care costs.

**Results:**

The cohort consisted of 693 383 patient-trials with 26 649 (3.8%) enrolling in SSVF (mean [SD] age, 52.7 [12.6] years; 89.6% male) and 666 734 (96.5%) in the no SSVF group (mean [SD] age, 53.8 [13.0] years; 90.8% male). Enrollment in SSVF was associated with a decrease in the risk of mortality (hazard ratio, 0.87; 95% CI, 0.82-0.92). In addition, enrollment in SSVF was associated with an increase in outpatient costs ($7534; 95% CI, $6767-$8302) and a decrease in inpatient costs (−$10 020; 95% CI, −$13 644 to −$6396).

**Conclusions and Relevance:**

In this study, federal prevention solutions to homelessness were associated with improved health outcomes and lower inpatient costs, which should inform national policy debates within and beyond the VA.

## Key Points

**Question:**

Is the Supportive Services for Veteran Families (SSVF) program associated with reduced health care costs and risk of mortality for veterans experiencing housing instability?

**Findings:**

This cohort study using a targeted trial emulation approach included 693 383 patient-trials comprising 229 096 unique patients. Compared with those not enrolled in SSVF, veterans enrolled in SSVF had significantly lower inpatient costs and mortality rates.

**Meaning:**

In this study, rapid rehousing and homelessness prevention initiatives may have important effects on health and health care utilization metrics.

## Teaser

This cohort study uses a target trial emulation approach to estimate the effect of enrolling in Supportive Services for Veteran Families on mortality and health care costs in the Veterans Affairs health care system.

## Introduction

Research demonstrates strong links between housing stability and health, with homelessness associated with cardiovascular disease,^1,2^ diabetes complications,^3,4,5,6^ substance use,^7,8^ and traumatic injuries.^9,10^ Given the multiple potential pathways for homelessness to impact health, it is not surprising that homelessness correlates with excess health care costs^11^ and mortality.^12^

The Federal Strategic Plan to Prevent and End Homelessness, released by the US Interagency Council on Homelessness, describes 3 types of homelessness prevention.^13^ Primary prevention includes protective interventions focused on housing security for high-risk populations. Secondary prevention involves helping individuals stabilize in housing without needing more intensive and long-term services. Tertiary prevention focuses on rehousing and stabilization of individuals who are no longer in stable housing.

While evidence suggests that permanent supportive housing (ie, long-term rental support for individuals experiencing housing insecurity) can be effective at improving housing stability,^14^ less is known regarding short-term assistance interventions with a prevention focus. Evidence of the effectiveness of temporary financial assistance (TFA) comes from observational data in Chicago, Illinois, from 2010 to 2012^15^ and a recent randomized clinical trial (RCT) conducted in Santa Clara, California.^16^ Both studies found that TFA had a significant impact on stable housing outcomes.

The Department of Veterans Affairs (VA) Supportive Services for Veteran Families (SSVF) program offers primary prevention through homelessness prevention services for those at imminent risk and rapid rehousing services for veterans who are currently homeless (eg, secondary prevention and tertiary prevention).^17^ SSVF offers case management, outreach, assistance in obtaining both VA and non-VA benefits as well as TFA, similar to the Chicago and Santa Clara interventions.

To date, the TFA component of SSVF has been shown to be associated with lower health care costs,^18,19^ improved health outcomes (including lower risks of suicidal ideation and mortality),^20^ and improved short-term^21^ and intermediate-term housing outcomes.^22^ However, SSVF operates as a package of services and assistance that vary according to the needs of the client. Using a target trial emulation approach, a framework to closely approximate an RCT to minimize bias when only observational data are available, a recent study found long-term improvements in housing stability for veterans who did and did not enroll in SSVF.^23^ However, to our knowledge, no studies have specifically evaluated the overall effects of SSVF in health and health care cost outcomes. And more broadly, outside VA, few studies have examined the impact of rapid rehousing and homelessness prevention services on health and health care costs.^24^ One study found improved mental health outcomes among a cohort of 98 single adults following placement in a rapid rehousing program.^25^

The objective of this study was to estimate the effect of enrolling in SSVF on mortality and health care costs in the VA health care system. Using data from the VA electronic health record, we identified veterans experiencing housing instability and constructed a series of target trial emulations evaluating the effect of SSVF on mortality and health care costs for 3 years following documentation of unstable housing.

## Methods

This study followed the Strengthening the Reporting of Observational Studies in Epidemiology (STROBE) reporting guideline. This cohort study using a target trial emulation approach was approved by the University of Utah Institutional Review Board. The Institutional Review Board granted a waiver of informed consent because this was a minimal-risk retrospective study using existing clinical and administrative data, and obtaining consent from all individuals was not practical. All procedures followed were in accordance with the ethical standards of the responsible committee on human experimentation and with principles of the Declaration of Helsinki.^26^

### Setting

The SSVF program is administered through community-based nonprofit organizations (referred to as grantees) with funding from the VA. Grantees may provide a variety of services tailored to household needs: (1) outreach to the community and within VA, (2) case management, (3) assistance obtaining VA benefits, (4) assistance in obtaining non-VA benefits, and (5) TFA consisting of financial assistance with rent, utility payments, security deposits, and other housing-related expenses.

### Data

We used the VA Corporate Data Warehouse to identify patients each month from October 2015 to December 2018 with housing instability evident in structured or unstructured data and as a source of baseline covariates. Data were analyzed from November 1, 2023, to September 9, 2025. Mortality data were obtained from the Death Ascertainment File, which includes death dates identified in the Corporate Data Warehouse as well as the US Social Security Administration Death Master File. While death dates are available from the Death Ascertainment File, cause of death is not. The cost of providing care for patients in VA facilities from the VA perspective is captured through an activity-based accounting system and made available to researchers in the VA Managerial Cost Accounting datasets. Types of health care services for which cost data are available in Managerial Cost Accounting data include outpatient, inpatient, emergency department, laboratory, imaging, and pharmacy, among others. Finally, SSVF enrollment was captured in Veteran-level Homeless Management Information System data used by grantees to track SSVF utilization.

### Target Trial Emulation Study Design

For a study examining the impact of SSVF on patient outcomes, the ideal comparison group would be persons not enrolling in SSVF who have similar demographic, comorbidities, and homelessness experience as those who do. Additionally, an accurate assessment of outcomes between veterans who do and do not enroll in SSVF should follow these individuals over a similar time period. While SSVF enrollment serves as a logical starting point for this follow-up period for those who enroll in SSVF, a similar time zero does not exist for veterans in the comparison group. The target trial emulation approach overcomes both the challenges of identifying a suitable comparison group as well as designating a time zero for that comparison group.^12,13,14,15^ In a target trial emulation, investigators hypothesize an RCT that could be conducted to answer the causal question of interest. Each element of this RCT is then replicated using observational data, modeled as a series of nested trials, with a separate trial for each month from January 2016 to December 2018. This nesting by month mitigates noncomparable start points for those not enrolling.

eTable 1 in Supplement 1 describes the elements of our target RCT along with the trial emulation analog using observational data. Additional details are described by Chapman et al.^23^

#### Eligibility Criteria

Inclusion for the hypothetical target RCT required qualifying for housing instability (homeless or at risk, based on structured or unstructured data), age 18 years or older, receipt of care in the VA system, and no prior experience with SSVF. The emulated trial would have similar eligibility criteria.

We implemented an operational definition of housing instability that required the presence of 2 types of evidence in the month prior to the trial start. The first was a structured data element indicating homelessness such as an *International Statistical Classification of Diseases and Related Health Problems, Tenth Revision* diagnostic code, a positive homelessness screening response, a clinic code (referred to as a stop code) indicating that the encounter occurred in a clinic providing homeless-related services, or administrative data recorded in the VA Homeless Outcomes Management and Evaluation System.^27^ Second, documentation of housing instability in free-text clinical notes extracted using a previously validated natural language processing system.^28^

Veterans who were enrolled in the no SSVF group in 1 month’s emulated trial remained eligible for future trial months with potential to enroll in either the no SSVF or the SSVF group during those later trials. In other words, veterans could be included in multiple trials. In our outcome models, we used an intent-to-treat approach by considering each veteran according to the treatment group they were initially assigned for that particular trial.

#### Treatment Assignment

Veterans who enroll in our hypothetical target RCT would be assigned to either a treatment or control group through a randomization process. Veterans assigned to the treatment group would enroll in SSVF, with access to TFA, case management, and other services. Veterans assigned to the control group would receive usual care, which may consist of housing support services offered both in the VA and the community.

In our emulated trial, a subset of veterans meeting eligibility criteria enrolled in SSVF each month. This enrollment was not randomly assigned but was correlated with a variety of veteran characteristics. We assumed that treatment assignment is random, conditional on observed baseline factors recorded in the Corporate Data Warehouse prior to the start date of each trial in which the patient enrolled. These baseline factors included demographics such as sex, race and ethnicity, and age; rurality; distance from the closest VA facility; Charlson Comorbidity Index; service-related disability; mental health or substance use disorder; enrollment in other VA homelessness programs; prior health care costs (specifically, costs in quarter −1 and −4); which type of structured data indicated housing instability in the month prior to enrollment; time since first structured documentation of homelessness; the count of housing-related visits in the last year; and the proportion of those visits classified by natural language processing as unstable. Race and ethnicity data were obtained from self-report in the VA electronic health record data (Corporate Data Warehouse).

For each month from January 2016 through December 2018, if eligible veterans enrolled in SSVF, they were assigned to the SSVF group for the emulated trial corresponding to that month. Otherwise, they were assigned to the no SSVF group. Time zero for each emulated trial was defined as the first day of that month.

#### Follow-Up and Comparison of Outcomes

For the hypothetical target RCT, enrollees would be followed up for 3 years after enrollment, with data on mortality and health care costs in the VA health care system collected by research staff.

For patients who enrolled in at least 1 emulated trial, death dates and health care costs in the VA health care system that occurred over the 3-year follow-up period were captured in VA administrative data. We examined total health care costs as well as costs separated into inpatient, outpatient, and pharmacy services. Following an intent-to-treat approach, we did not censor patients who crossed over from no SSVF.

### Statistical Analysis

We calculated descriptive statistics for key characteristics among patients enrolled in SSVF or no SSVF trials. In addition, we constructed Kaplan-Meier curves for our mortality outcome and unadjusted means for our health care cost outcomes.

To adjust for differences in baseline characteristics between SSVF and no SSVF patient-trials, we performed inverse probability of treatment weighting.^19^ For each patient-trial, we modeled the probability of enrolling in SSVF dependent on baseline characteristics using a logistic regression model that included fixed effects for each of the 36 trials.

Inverse probability of treatment weighting values, used to create the pseudopopulation, were calculated as 1 over the predicted probability that a patient-trial would enroll in the patient’s observed treatment group. Weights were stabilized using the proportion of patient-trials that enrolled in SSVF during a particular trial.

The outcome model for mortality was a Cox proportional hazards regression. As alternative specifications, we also ran accelerated failure time models using Weibull and exponential distribution assumptions. For health care costs, we estimated generalized linear models.^29,30^ We used the modified Park test which indicated that a γ distribution and a log link function was the most appropriate specification. For instances in which more than 20% of observations were 0, we used a 2-part model. The first part of this 2-part model was a logistic regression while the second part was a generalized linear models with a γ distribution and a log link function. For each outcome model, we calculated clustered standard errors within veterans and included fixed effects for each trial.

Given that we have observational data, we used the approach of VanderWeele and Ding^31^ to estimate an E-value for our mortality outcome. An E-value is the minimum strength of an association on the risk ratio scale that an unmeasured confounder would need to have with both the treatment and the outcome, conditional on measured covariates, to explain the estimated treatment effects reported here. Data were analyzed using Stata version 18 (StataCorp). Statistical significance was set at 2-sided *P* < .05.

## Results

The cohort consisted of 693 383 patient-trials with 26 649 (3.8%) enrolling in SSVF (mean [SD] age, 52.7 [12.6] years; 89.6% male) and 666 734 (96.5%) in the no SSVF group (mean [SD] age, 53.8 [13.0] years; 90.8% male). These 693 383 patient-trials comprised 229 096 unique patients. Descriptive statistics of baseline characteristics are shown in Table 1.

**Table 1.** Descriptive Statistics

Characteristic	Count (%)					
	Unweighted			Weighted		
	SSVF (n = 26 649)^a^	No SSVF (n = 666 734)^a^	SMD	SSVF (n = 26 649)^a^	No SSVF (n = 666 734)^a^	SMD
**Demographics**						
Age, mean (SD), y	52.7 (12.6)	53.8 (13.0)	0.08	52.8 (12.8)	52.7 (13.0)	0.01
Sex						
Female	2759 (10.4)	61 508 (9.2)		2759 (10.4)	61 508 (9.2)	
Male	23 890 (89.6)	605 226 (90.8)	0.04	23 890 (89.6)	605 226 (90.8)	0.04
Race and ethnicity^c^						
Black	10 381 (39.0)	238 648 (35.8)	0.07	9722 (36.5)	241 358 (36.2)	0.01
Hispanic	1802 (6.8)	42 754 (6.4)	0.01	1802 (6.4)	42 871 (6.4)	0.00
White	13 996 (52.5)	374 204 (56.1)	0.07	14 905 (55.9)	376 238 (56.4)	0.01
Other/missing^d^	2030 (7.6)	48 660 (7.3)	0.01	2023 (7.6)	49 138 (7.4)	0.01
Rurality	3213 (12.1)	109 186 (16.4)	0.12	3213 (13.5)	91 143 (13.7)	0.01
**Comorbidities and health care utilization**						
Charlson Comorbidity Index, mean (SD)	1.3 (2.0)	1.4 (2.1)	0.09	1.4 (2.1)	1.4 (2.1)	0.02
Any mental health diagnosis	18 696 (70.2)	508 640 (76.3)	0.14	18 696 (76.2)	507 118 (76.1)	0.00
Any substance use disorder	13 625 (51.1)	373 018 (55.9)	0.10	13 625 (56.2)	371 838 (55.8)	0.01
Outpatient costs, $^b^	14 083 (15 038)	15 178 (16 334)	0.07	14 956 (15 621)	15 140 (16 314)	0.01
Inpatient costs, $^b^	14 663 (39 744)	20 638 (52 464)	0.13	21 352 (58 680)	20 417 (51 982)	0.02
Pharmacy costs, $^b^	1706 (7755)	1869 (8525)	0.02	1819 (7595)	1863 (8546)	0.01
**Homelessness history**						
Other VA homelessness programs						
HUD-VASH	6948 (26.1)	153 474 (23.0)	0.07	6948 (20.7	154 216 (23.1	0.06
GPD	4422 (16.6)	83 024 (12.5)	0.12	4422 (14.2)	84 075 (12.6)	0.05
Months since first documentation of housing instability, mean (SD)	39.7 (31.3)	44.9 (32.1)	0.16	43.0 (31.1)	44.7 (32.1)	0.05
No. of visits with natural language processing documentation of housing status, mean (SD)^b^	19.1 (19.2)	20.1 (20.3)	0.05	20.2 (20.4)	20.1 (20.2)	0.01
Proportion of visits classified as unstable, mean (SD)	0.77 (0.19)	0.72 (0.24)	0.24	0.73 (0.21)	0.72 (0.24)	0.06

Abbreviations: GPD, grants and per diem; HUD-VASH, US Department of Housing and Urban Development-VA Supportive Housing; SMD, standardized mean difference; SSVF, Supportive Services for Veteran Families; VA, Veterans Affairs.

^a^ Number of patient-trials.

^b^ In year prior.

^c^ Race and ethnicity were obtained from self-report in the VA electronic health record data (Corporate Data Warehouse).

^d^ No further information about this classification is available.

**Figure 1.**
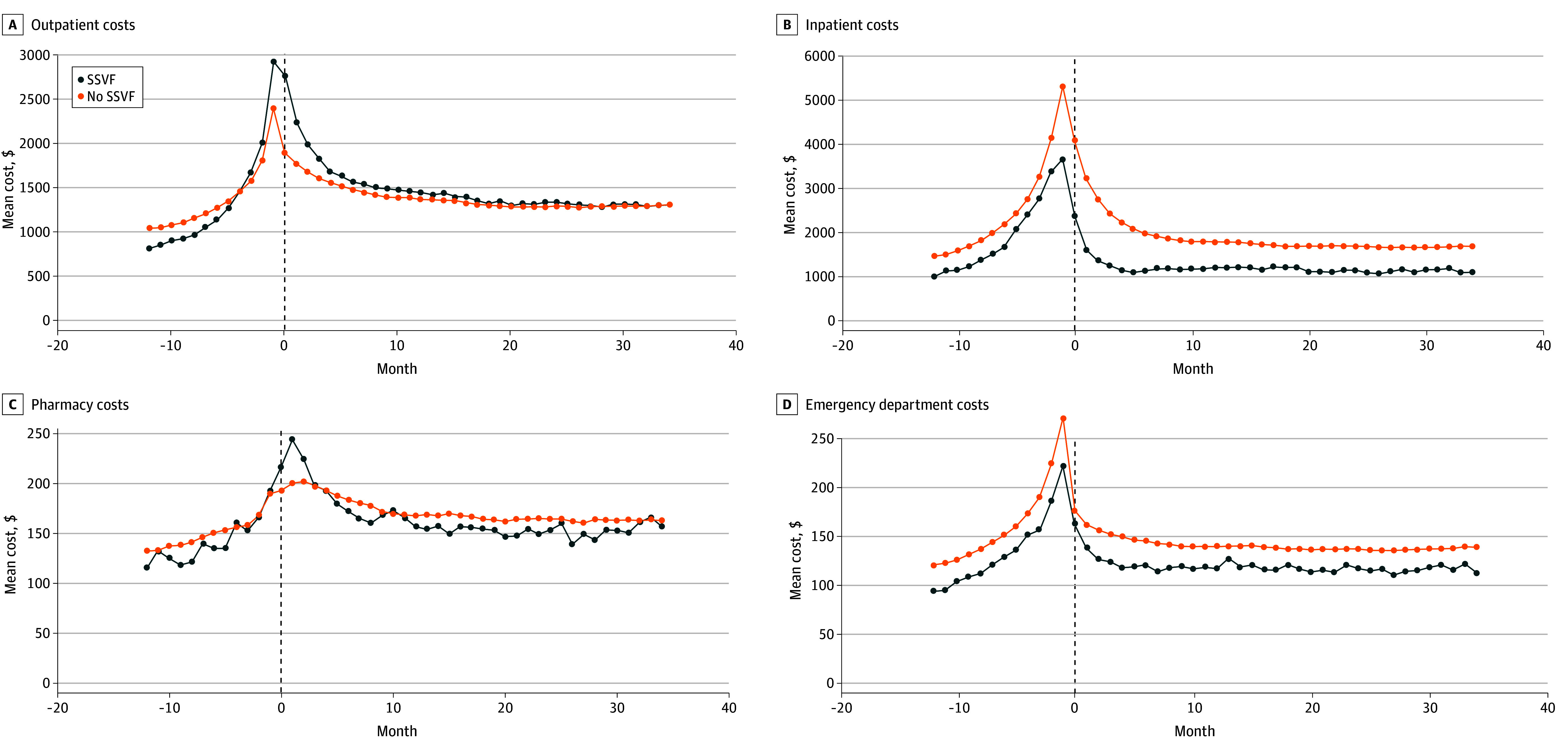
Dot Plot of Unadjusted and Unweighted Mean Costs Per Month for Patients in the Supportive Services for Veteran Families (SSVF) and No SSVF Groups for the 1 Year Preceding and the 3 Years Following the Index Date

**Figure 2.**
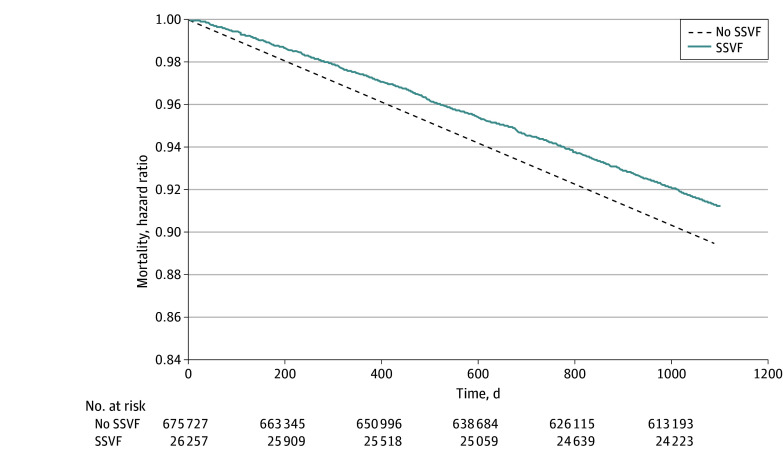
Kaplan-Meier Survival Curves for Supportive Services for Veteran Families (SSVF) and No SSVF Over the 3 Years Following the Index Date

Figure 1 shows unweighted mean health care costs per month. For both groups, mean health care costs increased prior to trial enrollment, peaking around the time of trial start, and decreased over the subsequent 3-year follow-up period. Weighted mean costs per month are shown in eFigure 1 in Supplement 1 and unweighted and weighted mean total costs per month are shown in eFigure 2 in Supplement 1. Kaplan-Meier curves demonstrated that the probability of survival was higher for those in the SSVF group consistently over the follow-up period (Figure 2).

In our inverse probability of treatment weighting analyses (eTable 2 in Supplement 1), SSVF was associated with reduced mortality over the 3-year follow-up period (hazard ratio [HR] = 0.87; 95% CI, 0.82-0.92) using a Cox proportional hazards regression (Table 2). Effect estimates from accelerated failure time models were nearly identical. In addition, outpatient costs over the 3-year follow-up period were $7534 (95% CI, $6767-$8302) higher for the SSVF group compared with the no SSVF group while inpatient costs were $10 020 (95% CI, $6396-$13 644) lower. There was no difference in emergency department, pharmacy, or total costs.

**Table 2.** Results From Inverse Probability of Treatment Weighted Regression Models

Outcome	Effect estimate (95% CI)	*P* value
Mortality^a^	0.87 (0.82 to 0.92)	<.001
Outpatient costs, $^b^	7534 (6767 to 8302)	<.001
Emergency department costs, $	77 (−92 to 246)	.37
Inpatient costs, $	−10 020 (−13 644 to −6396)	<.001
Pharmacy costs, $	240 (−38 to 517)	.09
Total costs, $	−774 (−4408 to 2860)	.68

^a^ Mortality outcome was analyzed using a Cox proportional hazards regression with effects measured as hazards ratios.

^b^ Cost outcomes were analyzed using a generalized linear models and 2-part models with effects measured as marginal effects.

**Figure 3.**
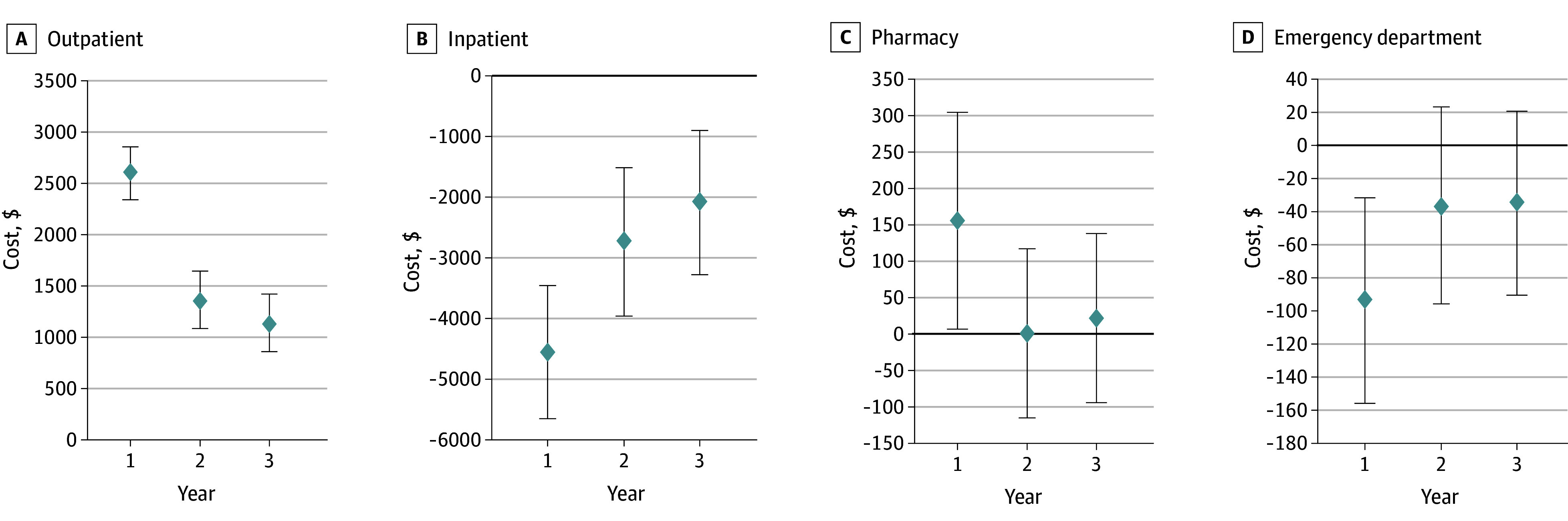
Dot Plot of Year-Specific Marginal Effects From Inverse Probability of Treatment Weighted Regression Models for Health Care Cost Outcomes Error bars indicate 95% CIs.

Year-specific marginal effects for costs show that the largest absolute values occurred in year 1 (Figure 3). Outpatient costs were $4063 (95% CI, $3769-$4357) higher in the SSVF group compared with the no SSVF group in the first year but were $1985 (95% CI, $1658-$2312) higher in year 2 and $1494 (95% CI, $1155-$1833) higher in year 3. Similarly, SSVF was associated with $4724 (95% CI, $3515-$5933) lower inpatient costs in the first year and this decrease was smaller in years 2 (marginal effect = $2322; 95% CI, $909-$3735) and 3 (marginal effect = $2838; 95% CI, $1269-$4407).

The point estimate E-value for the mortality outcome was 1.581 while the lower bound E-value was 1.401. This means that an unmeasured confounder would need to be associated with both mortality and enrollment in SSVF by an HR of at least 1.581 each to explain our estimated HR and by at least 1.401 to shift the 95% CI to include the null.

## Discussion

SSVF is one of the largest homeless assistance programs in the US. Using a target trial emulation approach, we estimated that SSVF would be associated with reduced risk of mortality and lead to an increase in outpatient costs along with a decrease in inpatient costs during the 3 years after initiating SSVF. The effects in health care costs were strongest in the first year, which coincides with receipt of SSVF services for those in the treatment group. It is noteworthy that these effects persist, albeit smaller magnitude, for 3 years given that SSVF is designed for short-term assistance. This sustained, but dampened, effect is consistent with a previous study^23^ by some authors of the present study that found improved housing outcomes over the 3-year follow-up period. These results are also consistent with previous studies^32^ that have found that supportive housing programs are associated with increases in outpatient use and decreases in inpatient use.

Our findings of an estimated 13.5% reduction in mortality risk associated with SSVF may have additional relevance because the mortality rate of unhoused individuals is 3 to 4 times higher than that in the general population.^33^ In a recent study documenting the rapid rise in mortality rates among unhoused individuals in the US, the authors suggest that, “the most effective form of mortality prevention is preventing occurrence of homelessness in the first place and rehousing people experiencing homelessness as quickly and stably as possible.”^34^ Our current findings provide strong support for this statement and are consistent with those found in similar studies.^35,36^

This study may have implications that extend beyond veterans based on several recent developments and policies at the intersection of housing and health. The first is the increased investment in housing made by health care payers and systems over the past decade.^37^ For instance, 1 study found that, between 2017 and 2019, 57 health systems representing more than 900 hospitals in the US invested $1.6 billion in housing programs.^38^ Second, in December 2022, the Center for Medicare & Medicaid Services announced that the Medicaid program would offer waivers (Section 1115 waivers) to expand the tools available to states to address social determinants of health, including housing and food security.^39^ Besides providing some insight into health benefits that could result from these new Medicaid-covered programs, our findings are especially relevant because of requirements that Section 1115 waivers must be budget neutral, ie, not lead to net increases in costs to CMS.^40^ Finally, the US Interagency Council on Homelessness recently released the first homelessness prevention framework by the federal government.^41^ Our findings suggest that large-scale efforts to prevent homelessness in the US may lead to substantial improvements in survival and lower inpatient costs.

To date, few studies have documented the impact of housing interventions on health care costs in the US. Our team found that TFA was associated with decreases in inpatient and total health care costs^18^ along with increases in primary care and mental health outpatient costs^19^ among SSVF enrollees. Beyond previous work by authors of the present study,^18,23^ there has been virtually no attempt to assess the impact of homelessness prevention and rapid rehousing interventions on similar outcomes. Several other studies have focused on the effect of permanent supportive housing, which offers longer-term housing for people experiencing homelessness, on health care costs Massachusetts^42^ and Denver, Colorado.^43^ Findings from those studies are generally consistent with our findings, suggesting a potential benefit for housing interventions for health and health care costs.

SSVF is comprised of multiple components beyond just TFA. The similarities between our current findings and those from previous analyses of both health care costs^18,19^ and mortality^20^ might suggest TFA as the driving force behind these results. However, future analyses that examine the relative contribution of each SSVF component would be helpful for housing advocates and policymakers designing similar programs.

### Limitations

This study has several limitations. First, because the SSVF program is specific to the US veteran population, caution is recommended before generalizing our findings to other groups of unhoused individuals. Second, while death dates were obtained regardless of setting, we included only health care costs incurred within the VA health care system. Third, as is the case with any observational study, our analysis is subject to residual confounding. There are a variety of reasons why veterans experiencing housing instability might enroll in SSVF. On one hand, it could be those who are more proactive and motivated to find a solution to their housing struggles. On the other hand, it could be that SSVF grantees purposely identify veterans who have the most barriers to stable housing and who may have the most to gain from the services provided through SSVF. In our analytical approach, we controlled for a number of measurable characteristics from VA electronic health record data. However, future studies should seek to better understand the reasons for SSVF enrollment, which may shed light on additional control variables to include in subsequent analyses.

## Conclusions

SSVF is one of the largest homelessness programs in the country with participants in every state in the US. This cohort study using a target trial emulation approach found that SSVF was associated with improved health outcomes and with lowering inpatient costs.

## Notes

Supplement 1. **eFigure 1.** Unadjusted, weighted mean costs per month for patients in the SSVF and no SSVF groups for the 1 year preceding and the 3 years following the index date: outpatient, inpatient, pharmacy, and emergency department costs **eFigure 2.** Unadjusted mean total costs per month for patients in the SSVF and no SSVF groups for the 1 year preceding and the 3 years following the index date **eTable 1.** Target trial and emulation trial elements **eTable 2.** Descriptive statistics for inverse probability of treatment weights by treatment group

Supplement 2.
Data Sharing Statement
