# Supplementary material for: Health Care Outcomes of Homelessness Prevention Programs in Veterans Experiencing Housing Instability
Source: JAMA Health Forum. 2026 Jan 23;7(1):e256417. doi: 10.1001/jamahealthforum.2025.6417 (PMC12831159; doi:10.1001/jamahealthforum.2025.6417)
Supplement: Supplement 1. — eFigure 1. Unadjusted, weighted mean costs per month for patients in the SSVF and no SSVF groups for the 1 year preceding and the 3 years following the index date: outpatient, inpatient, pharmacy, and emergency department costs eFigure 2. Unadjusted mean total costs per month for patients in the SSVF and no SSVF groups for the 1 year preceding and the 3 years following the index date eTable 1. Target trial and emulation trial elements eTable 2. Descriptive statistics for inverse probability of treatment weights by treatment group [file jamahealthforum-e256417-s001.pdf]

Supplemental Online Content

Nelson RE, Chapman AB, Montgomery AE, et al. Health care outcomes of homelessness prevention programs in veterans experiencing housing instability. *JAMA Health Forum.* 2026;7(1):e256417. doi:10.1001/jamahealthforum.2025.6417

eFigure 1. Unadjusted, weighted mean costs per month for patients in the SSVF and no SSVF groups for the 1 year preceding and the 3 years following the index date: outpatient, inpatient, pharmacy, and emergency department costs

eFigure 2. Unadjusted mean total costs per month for patients in the SSVF and no SSVF groups for the 1 year preceding and the 3 years following the index date

eTable 1. Target trial and emulation trial elements

eTable 2. Descriptive statistics for inverse probability of treatment weights by treatment group

This supplemental material has been provided by the authors to give readers additional information about their work.

© 2026 Nelson RE et al. *JAMA Health Forum*.

eFigure 1: Unadjusted, weighted mean costs per month for patients in the SSVF and no SSVF groups for the 1 year preceding and the 3 years following the index date: outpatient, inpatient, pharmacy, and emergency department costs

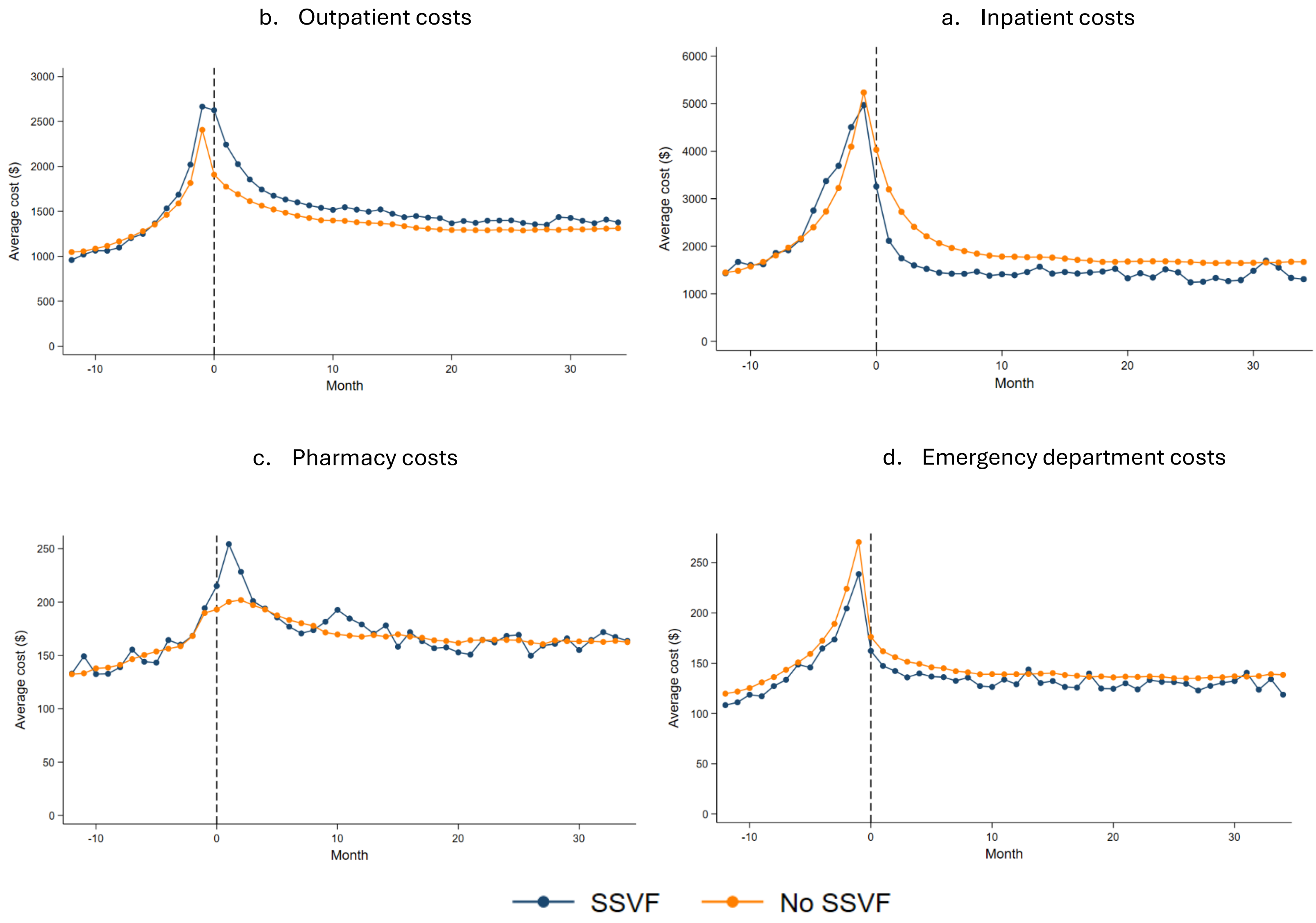

© 2026 Nelson RE et al. *JAMA Health Forum*.

eFigure 2: Unadjusted mean total costs per month for patients in the SSVF and no SSVF groups for the 1 year preceding and the 3 years following the index date

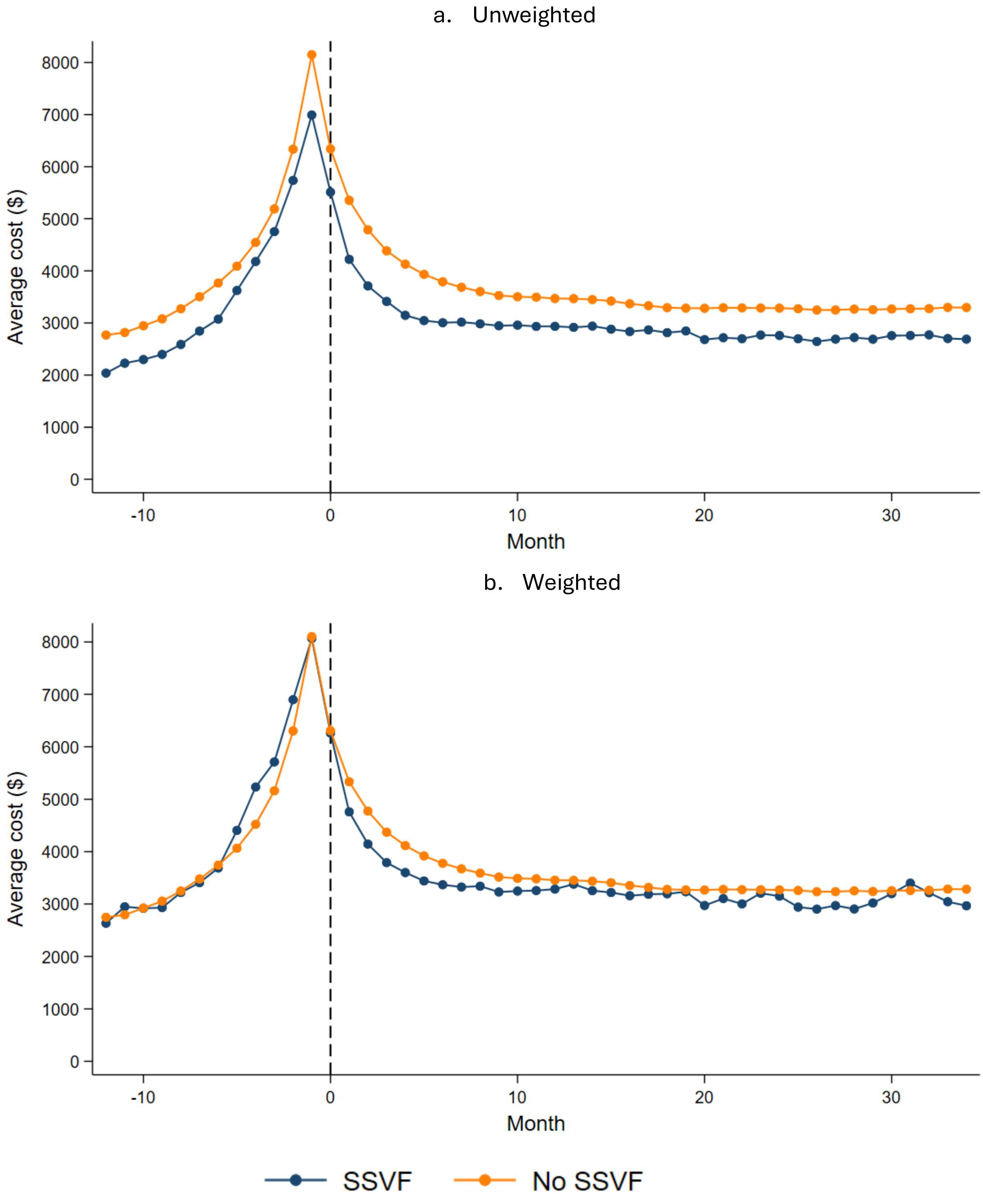

© 2026 Nelson RE et al. *JAMA Health Forum*.

eTable 1: Target trial and emulation trial elements

Trial element and description	Hypothetical target trial	Emulation using observational data
1. Units and eligibility criteria	Veterans experiencing housing instability.	Veterans with encounters in the VA healthcare system with documentation of housing instability using both structured EHR data elements (e.g., ICD-10 codes) and in free-text clinical notes.
2. Definitions of exposure and comparison conditions	Treated Veterans would be enrolled in SSVF;<br>Untreated Veterans would receive usual care, consisting of housing support services offered both in the VA and the community. These interventions span the three levels of public health prevention from universal screening (primary), to transitional housing (secondary), to permanent supportive housing (tertiary).	Treated Veterans would be enrolled in SSVF;<br>Untreated Veterans would receive usual care, consisting of housing support services offered both in the VA and the community. These interventions span the three levels of public health prevention from universal screening (primary), to transitional housing (secondary), to permanent supportive housing (tertiary).
3. Assignment mechanism	Randomization	Randomization is emulated using a nested trial design and inverse probability of treatment weights (IPTW)
4. Baseline and follow-up	Baseline = time of randomization<br>Follow-up = 3 years post-randomization	Baseline = time of randomization<br>Follow-up = 3 years post-treatment assignment
5. Outcomes	Mortality and healthcare costs from the VA's perspective	Mortality and healthcare costs from the VA's perspective
6. Causal estimand	Intent-to-treat<br>Expected estimand: the effect of being assigned to SSVF	Intent-to-treat<br>Expected estimand: the effect of being assigned to SSVF
7. Statistical analysis	Cox proportional hazards regression and generalized linear models	IPTW Cox proportional hazards regression and generalized linear models with fixed effect for each trial

© 2026 Nelson RE et al. *JAMA Health Forum*.

eTable 2: Descriptive statistics for inverse probability of treatment weights by treatment group

Statistic	No SSVF	SSVF
Mean	1.000087	0.987481
Standard deviation	0.019480	0.734438
Minimum	0.966150	0.338166
25th percentile	0.987896	0.634467
Median	0.999099	0.832013
75th percentile	1.011347	1.108362
Maximum	1.316164	22.545460

© 2026 Nelson RE et al. *JAMA Health Forum*.
