# Supplementary material for: Health Care Outcomes of Homelessness Prevention Programs in Veterans Experiencing Housing Instability
Source: JAMA Health Forum. 2026 Jan 23;7(1):e256417. doi: 10.1001/jamahealthforum.2025.6417 (PMC12831159; doi:10.1001/jamahealthforum.2025.6417)
Supplement: Supplement 2. — Data Sharing Statement [file jamahealthforum-e256417-s002.pdf]

Data Sharing Statement

Nelson. Health Care Outcomes of Homelessness Prevention Programs in Veterans Experiencing Housing Instability. *JAMA Health Forum*. Published January 23, 2026. doi:10.1001/jamahealthforum.2025.6417

Data
Data available: No

Additional Information
Explanation for why data not available: Aggregate data will be made available, but the VA does not allow posting of administrative data
